# Valorisation of Biomass Waste for Sustainable Bioenergy and Biofuel Production

**DOI:** 10.3390/bioengineering10050619

**Published:** 2023-05-21

**Authors:** Pei-Ti Sun, Huadong Peng

**Affiliations:** 1 Department of Bioengineering and Imperial College Centre for Synthetic Biology, Imperial College London, London SW7 2AZ, UK; 2 The Novo Nordisk Foundation Center for Biosustainability, Technical University of Denmark, 2800 Kongens Lyngby, Denmark

## 1. Introduction 

Although the rapid development of industrialisation has brought great benefits to our societies, waste accumulation and energy depletion have inevitably grown to be critical issues in recent decades. Biomass wastes from a wide range of sources, including household leftovers, agricultural, forestry, and industries [1], are among the scientific community’s gravest concerns today. These wastes can be disposed of by either landfill or traditional combustion. However, disposal uses a large amount of land and results in greenhouse gas emissions. Hence, it is critical to investigate the correct ways of managing or re-utilizing the biowaste and ultimately pursuing a green economy. For example, biomass wastes could be used to serve as alternative feedstocks, providing green and sustainable biofuels such as bioethanol, biogas, and biodiesel [2]. Cellulose-rich crop residues like wheat straw and corn stover are common agricultural wastes that possess potential uses as feedstock for bio-refinery. Digestate wastewater, poultry litter, and animal manure are also versatile sources of bioenergy and nutrients for microbial growth due to their abundance levels of nitrogen and trace elements. Microorganisms including yeasts, algae and bacteria generally participate in the utilisation of biomass wastes. With the rapid development of synthetic biology-guided bioeconomy, superior microbial cell factories will be constructed to degrade the biowaste and produce value-added bioproducts [3]. Thus, the utilisation of biomass wastes is important in efforts to alleviate the waste burden and promises to reduce human reliance on non-renewable petrol energy resources. 

This editorial paper summarises papers published recently in *Bioengineering* (Basel) on the study of bioenergy and biofuels production using biomass waste as feedstocks. 

## 2. Renewable Bioethanol from Lignocellulose

Bioethanol and biogas are widely used as additives to fuels, and they are thought to have the potential to serve prospective energy sources to replace traditional fossil fuels in the green era. However, crops and sugar cane are mainly used in current processes of bioethanol production; this implies that feedstock availability would be limited by the costs of and competition over edible carbon sources. Therefore, it is necessary to search for relatively low-cost and non-food-competing materials for use in bioenergy production [4].

Lignocellulose, composed of lignin, cellulose, and hemicellulose, is proposed as a promising substrate for use in bioethanol production. For example, in one study, the lignocellulose-rich banana leaf waste was firstly pre-treated via the hydrothermal approach followed by enzymatic hydrolysis to yield 13.7 g/L and 18.4 g/L glucose from the slurry and solid mixtures, respectively [5]. Subsequently, ethanol production was achieved at the conversion efficiency of 94.6% from cellulose in pretreated solid fractions [5]. In another study, lignocellulosic biomass was found to contain wax on the surface to protect itself from external stresses; however, this probably affects saccharification, which is a critical step in the production process of bioethanol. Herein, wax interferences were then investigated to improve the saccharification efficiency. Research showed that dewaxing biomass resulted in increased sugar and ethanol yields with all tested lignocellulosic biomasses [6]. Furthermore, the extracted wax from raw biomass was analyzed by GC-MS. The researchers identified the major composition of fatty acid methyl esters and other valuable compounds, attributes that enhance its value on market and potential for future commercialization [6].

Microorganisms like bacteria and yeasts are widely used in the fermentation for bioethanol production, which plays a critical role in biomass waste utilisation. As several steps of biochemical reactions are required for hemicellulosic sugar to enter cells, sugar transportation and cofactor imbalance in yeast metabolism have been studied to achieve the efficient uptake of hemicellulose sugar [7]. To optimize yields, various metabolic strategies via genetic and proteomic engineering were developed for ethanol production from lignocellulose. For example, the overexpression of heterologous genes *pdc* and *adhB* can direct carbon flux to ethanol synthesis in *Escherichia coli* [8]. Direct ethanol production can also be achieved using a cellulolytic *Saccharomyces cerevisiae* strain optimized with protein folding capacity and enzyme secretion [9].

## 3. Sustainable Biodiesel and Lipid Production

Biodiesel, a biodegradable oil originating from plants, animals, and microorganisms, has become one of the hottest topics due to its potential for use in green energy. A bibliometric analysis exhibited trends to address sustainability challenges, along with a considerable amount of academic output regarding biodiesel production in this decade [10]. This study also specified that the research of biodiesel production is primarily carried out in Asia and America, where countries have similar economic interests [10].

Biodiesel can be used directly or obtained through several approaches, including pyrolysis, microemulsions, and mostly transesterification, where triglycerides react with alcohol. Since the presence of free fatty acids in raw materials lead to undesired saponification with alkali catalyst, it is crucial to select suitable catalysts under optimal conditions [11]. Despite being well-studied, homogenous catalysts still possess lots of disadvantages such as toxicity and flammability, and their use is accompanied by environmental concerns like wastewater. In comparison, biomass-derived catalysts are relatively stable and more renewable; however, enzymatic catalysts need further studies for commercialisation. Nano-catalysts and biowastes as feedstocks were likewise discussed, offering a novel route to improve current biodiesel production [12].

Apart from biocatalysts, metal–organic frameworks (MOFs) emerge as an alternative to making biofuels more accessible because of their adjustability, high surface area, tunable porosity, and versatility with functional groups and structures. Zr-MOFs (zirconium based) catalysts have been widely used and modified in order to synthesize various types of materials and fuels, though there are still challenges for industrial-scale applications [13].

Lipids constitute a typical type of biodiesel generally obtained from oil refineries and distribution networks. Being biodegradable and non-toxic, lipids are a prospective biofuel that can be produced from a variety of sources, making them a competitive energy source to petroleum fuels. In addition to sources in animal fats and plants oil, lipids can also be acquired from engineered microorganisms. Biosynthesis and metabolic strategies of fatty acids, as important precursors of lipids, were investigated to enable high-yield lipid production in yeast [14].

## 4. Pretreatment and Other Applications of Biowaste

Biomass wastes, as feasible feedstocks for use in biofuel and bioenergy production, usually consist of complex organics, polymetric substances, and inhibitory compounds, which may significantly hinder bioproduction process. To efficiently utilize biomass waste like lignocellulose, pretreatments, such as microwave irradiation, steam explosion, wet oxidation, and biochemical conversion, are applied in order to degrade complex chemical structures and stabilize carbohydrate formation [15]. For instance, microwave-assisted and ball milling pretreatments are potential approaches with which to enhance the enzymatic hydrolysis of microcrystalline cellulose, with no chemicals required [16].

Sludge from wastewater is another environmental issue, and its pretreatment methods are under investigation in order to improve biological treatments with anerobic or aerobic digestion [17]. To obtain the efficiency of degradability and biogas production from wastewater, the biophotocatalytic system was studied and optimized using a response surface methodology, revealing its potential for use in the treatment setting of effluent [18].

Wastewater is also ideal for use in algae cultivation due to its abundance in nutrition. Research shows that the cultivation of microalgae *Desmodesmus sp.* in anaerobic digestion wastewater boosted lipid production compared to the use of minimal media BG11. Moreover, defatted algae culturing in modified wastewater digestate led to increases in total biomass and valuable products [19]. Just like wastewater, chicken manure (CM) is an N-rich animal waste that can be used as cultivation media after anaerobic digestion treatment. Different dilutions of CM were tested for microalgae cultivation, and the results revealed the most suitable condition for *Chlorella vulgaris* CPCC 90 and provided a framework for further studies about bio-based byproducts and circular bioeconomy [20].

## 5. Conclusions

Biomass wastes have become among the most important prospective feedstocks for biofuels and bioenergy production in recent decades. Their use offers to not only address the waste accumulation issues and bring out alternative energy sources but also highlights the hot topic of bioenergy with carbon capture and storage (BECCS). In addition, the utilisation of biomass benefits local communities, particularly by boosting the economic growth in rural areas. Diverse approaches to and pretreatments of waste like lignocellulose and wastewater are currently under investigation to maximise their re-utilisation. Biotechnologies and engineering methods such as synthetic biology tools are also being applied to enhance the bioproduction of valuable products. However, further research is necessary to improve the efficiency of the entire process of biofuel production and ensure a carbon-neutral and sustainable future.
